# High-resolution analysis of DNA copy number alterations in patients with primary open-angle glaucoma

**Published:** 2009-08-15

**Authors:** Khaled K. Abu-Amero, Ali Hellani, Patrick Bender, George L. Spaeth, Jonathan Myers, L. Jay Katz, Marlene Moster, Thomas M. Bosley

**Affiliations:** 1 Ophthalmic Genetics Laboratory, Department of Ophthalmology, College of Medicine, King Saud University, Riyadh, Saudi Arabia; 2 PGD Laboratory, Saad Specialist Hospital, Al-Khobar, Saudi Arabia; 3 National Institutes of Mental Health, Rochville, MD; 4 William and Anna Goldberg Glaucoma Service, Wills Eye Hospital, Thomas Jefferson University Hospital, Philadelphia, PA; 5 Division of Neurology, Cooper University Hospital, Camden, NJ

## Abstract

**Purpose:**

To determine whether patients with isolated primary open-angle glaucoma (POAG) have evidence of chromosomal copy number alterations.

**Methods:**

Twenty-seven Caucasian and African-American POAG patients and 12 ethnically matched controls were carefully screened for possible glaucoma and tested for chromosomal copy number alterations using high resolution array comparative genomic hybridization.

**Results:**

No POAG patient had evidence of chromosomal copy number alterations when compared to normal ethnically matched controls. Additionally, there was no evidence of somatic mosaicism in any tested POAG patient.

**Conclusions:**

Chromosomal deletions and/or duplications were not detected in POAG patients as compared to controls. Other chromosomal imbalances such as translocations, inversions, and some ploidies cannot be detected by current array comparative genomic hybridization technology, and other nuclear genetic, mitochondrial abnormalities, or epigenetic factors cannot be excluded as a possible contributing factor to POAG pathogenesis.

## Introduction

Glaucoma is one of the leading causes of blindness worldwide [1] with a prevalence of over 2% in individuals older than 40 years [2]. Primary open-angle glaucoma (POAG) is the most common type of glaucoma in Western countries and has risk factors that include elevated intraocular pressure (IOP) and age [3], but these factors do not predict the presence or degree of visual loss [4]. Up to half of all patients with POAG have a positive family history, and the risk of POAG is increased three to nine times in first-degree relatives of POAG patients [2,5]. These observations suggest that genetic factors contribute to POAG [1,6,7].

Currently, 14 chromosomal loci are linked to POAG by the Human Genome Organization. Thus far, only three genes associated with POAG have been identified within these loci including myocilin [8], optineurin [9], and WD repeat domain 36 (*WDR36*) [10]. However, mutations in these three genes are present in less than 5% of POAG patients [11]. Over 20 other gene variants have been associated with the disorder, but in general, these loci have been identified from linkage analysis of family data sets often without corroboration by other investigators or in other populations. Therefore, the cause of the genetic risk for the occurrence of POAG remains largely unknown.

Several chromosomal aberrations have been reported to cause glaucoma, but in general, glaucoma has been associated with an obvious genetic syndrome in these patients [12,13]. To our knowledge, no study has investigated chromosomal copy number variations in patients with isolated POAG. Therefore, we examined possible chromosomal copy number changes in POAG patients using high resolution array comparative genomic hybridization (array CGH) technology.

## Methods

### Patients and controls

Patients were selected from the Glaucoma Clinic at Wills Eye Hospital (Philadelphia, PA) after examination by a glaucoma specialist and after obtaining informed consent approved by the Wills Institutional Review Board. This research adhered to the tenets of the Declaration of Helsinki, and all patients and controls signed an informed consent approved by the Wills Eye Hospital Institutional Review Board.

Patients were eligible for inclusion in this study if they met standard clinical criteria for POAG [14-16] including age greater than 40 years, IOP greater than or equal to 21 mmHg in at least one eye before treatment, normal-appearing anterior chamber angles bilaterally on gonioscopy, and optic nerve injury characteristic of POAG (with narrowed or absent rim, asymmetric cupping of the optic discs, and static visual fields compatible with optic disc appearance and with glaucoma). Exclusion criteria included historical, neuroimaging, or biochemical evidence of another possible optic neuropathic process affecting either eye, significant visual loss in both eyes not associated with glaucoma, or choosing not to participate.

All control subjects had full ophthalmologic examinations and static perimetry. Each had IOPs below 21 mmHg and symmetry in the two eyes, normal anterior chambers, optic discs that were normal and symmetric in appearance, entirely normal static perimetry (both eyes), and no prior history of glaucoma. All patients and controls had Humphrey Swedish interactive threshold algorithm (SITA) achromatic static perimetry, stimulus III, 24–2 (Humphrey Field Analyzer II; Carl Zeiss Meditec Inc., Dublin, CA).

### Array CGH technique

Blood was collected in acid-citrate-dextrose (ACD) tubes, and DNA was extracted using a Qiagen Autopure LS instrument (Qiagen, Valencia, CA) following the manufacturer’s recommended procedure. To detect chromosomal rearrangements, 2 μg of POAG patient genomic DNA was competitively hybridized with 2 μg of ethnically matched control DNA (as a reference sample) on an Agilent Human Genome CGH 244A Oligo Microarray Kit (Agilent Technologies Inc., Santa Clara, CA), which has an average probe spacing across the human genome of 6.4 Kb. Briefly, 50 μl of DNA from POAG patients and controls was digested using 50 units of Alu1 (Roche, Mannheim, Germany) and 50 units of Rsa1 (Roche) restriction enzymes in a 100 μl volume with 10 μl of 10X Promega Buffer C. Digestions were performed for 2 h at 37 °C. Digested samples were purified using QIAprep Spin Miniprep columns (Qiagen) and eluted according to the manufacturer's instructions. Samples were then analyzed using the Agilent 2100 Bioanalyzer with the DNA 7500 LabChip Kit and DNA 7500 Software Script (Agilent) as per the manufacturer’s instructions. Alu1/Rsa1 digested DNA samples were labeled using the BioPrime Array CGH Labeling Kit (Invitrogen, Carlsbad, CA) according to the manufacturer's protocol. POAG patient and control DNA samples were systematically labeled with Alexa Fluor 555 and 647, respectively.

Labeled products of each sample and control DNA were purified using QIAprep Spin Miniprep columns (Qiagen), mixed together, and checked on the Agilent 2100 Bioanalyzer (Agilent) to evaluate the Alexa Fluor 555 integration into the DNA samples. The following hybridization blocking reagents were added to the purified Alexa Fluor 555 and 647 labeled samples: 50 μg Cot-1 DNA (Invitrogen) and 50 μl of 10X control targets (Agilent). The volume was brought to 250 μl with double-distilled H_2_O, and 250 μl of 2X hybridization buffer (Agilent) was added. The hybridization mixture was then denatured at 100 °C for 3 min in a water bath. Samples were immediately transferred to a 37 °C water bath for 30 min to allow pre-annealing of the blocking agents to the labeled sample. Samples were centrifuged for 5 min at 16,000x g and immediately applied to the Agilent Human Genome CGH 244A Oligo Microarray Kit as per the manufacturer's recommendations. Hybridizations were performed at 65 °C for 42 h.

Microarrays were disassembled in Agilent wash buffer-1 at room temperature (RT), transferred to a slide holder, and incubated for 5 min with stirring in the Agilent wash buffer-1 at RT. The second washing step was performed for 1 min in wash buffer-2 at 37 °C. The third and fourth washing steps were done with acetonitrile (Fisher Scientific, Fair Lawn, NJ) and stabilization solutions (Agilent) for 1 min and 30 s at RT, respectively. Microarray slides were immediately scanned in the Agilent DNA Microarray Scanner using the default settings.

Data analysis was performed by Agilent Feature Extraction 9.1 and CGH Analytics 3.4 (Agilent). Log^2^ expression ratios were computed and normalized using CGH Analytics 3.4 software. Putative chromosome copy number changes were defined by intervals of three or more adjacent probes with log^2^ ratios suggestive of a deletion or duplication when compared with the log^2^ ratios of adjacent probes. The quality-weighted interval score algorithm (ADM2) was used to compute and assist in the identification of aberrations for a given sample.

**Figure 1:**
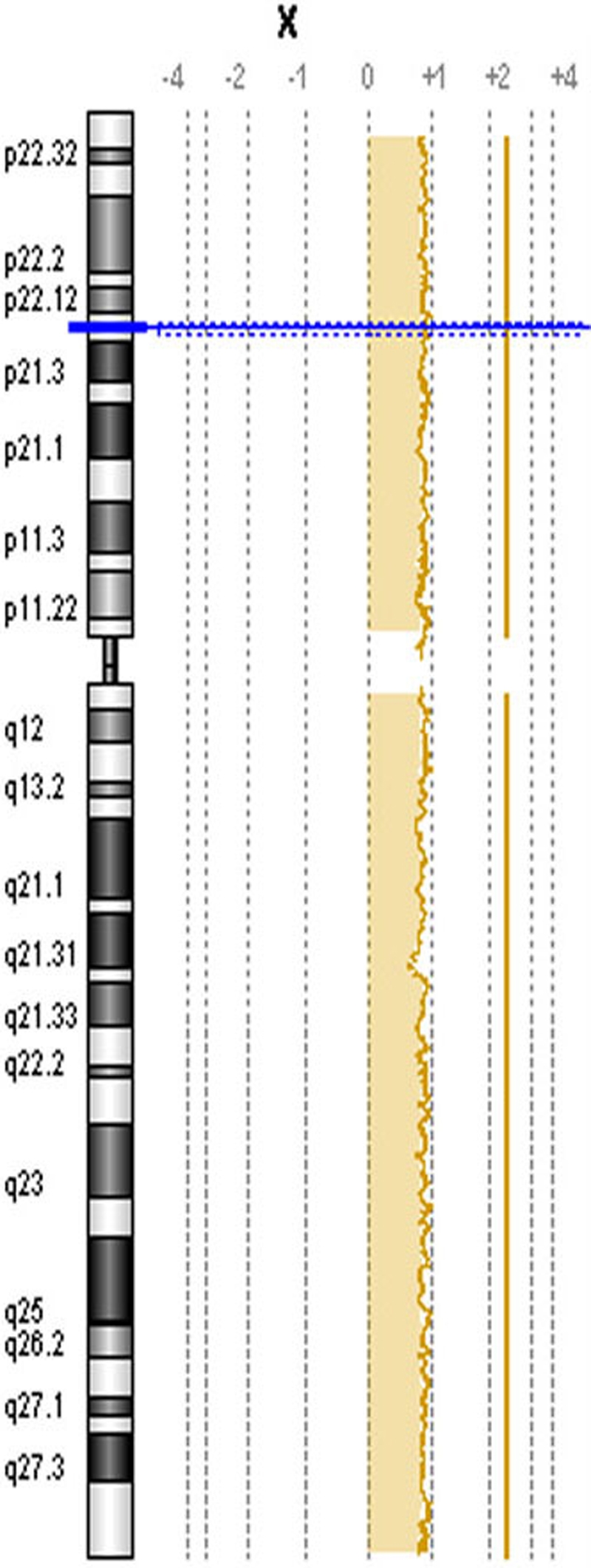
Array CGH result for internal control. As an internal quality control for the array CGH procedure, control DNA was hybridized against POAG DNA of the opposite sex (ratio of +1 with regard to chromosome X for XX POAG and XY control).

As an internal quality control measure, DNA from Caucasian patients were mixed with DNA from Caucasian controls of the same and opposite sex and co-hybridized to the 244K chip (Figure 1). The same was done for POAG patients and controls of African American ethnicity.

## Results

Clinical characteristics of the 27 POAG patients included in this study are detailed in Table 1. Patient sex (15 males and 12 females) and ethnicity (14 Caucasian and 13 African-American) were similar to that of the 12 controls, which were carefully screened for presence of POAG or other optic neuropathies (5 males and 7 females; 9 Caucasian and 3 African-American). The mean age of patients (70.7±10.8 years) was somewhat greater than that of controls (61.1±10.8 years).

**Table 1:** Clinical characteristics of POAG patients.

**Patient**	**Age**	**Sex**	**Race**	**VA OD**	**VA OS**	**Max IOP OD**	**Max IOP OS**	**Vertical C/D OD**	**Vertical C/D OS**	**HVF PSD OD**	**HVF PSD OS**
1	54	M	C	20/100	20/20	54	34	0.9	0.3	12.94	1.53
2	63	F	C	20/40	20/25	29	30	0.4	0.7	1.36	11.97
3	62	M	C	20/16	20/16	30	29	0.8	0.8	5.78	11.36
4	81	M	C	20/25	20/25	25	24	0.85	0.85	10.37	1.83
5	71	F	C	20/100	20/25	27	27	0.9	0.9	7.38	12.33
6	60	F	C	20/30	CF	35	26	0.9	0.9	12.37	5.37
7	79	M	C	20/20	20/20	28	21	0.85	0.65	2.18	1.89
8	97	M	C	HM	20/25	40	27	0.99	0.99	1.86	11.38
9	90	F	C	20/70	20/30	20	20	0.9	0.8	8.83	8.84
10	63	M	AA	20/25	20/20	25	25	0.8	0.85	1.46	3.8
11	64	F	C	20/30	20/20	23	21	0.9	0.9	11.78	10.49
12	66	M	AA	20/20	20/25	32	30	0.9	0.9	10.03	8.28
13	68	F	AA	20/20	20/25	30	30	0.5	0.6	1.27	1.99
14	75	F	C	20/400	20/30	25	25	0.95	0.8	10.63	3.73
15	81	F	C	20/70	20/25	30	20	0.8	0.65	4.62	1.91
16	76	M	AA	20/30	20/30	26	26	0.3	0.7	1.7	3.4
17	65	M	AA	20/30	20/50	29	31	0.85	0.8	1.81	1.98
18	84	M	C	20/20	20/20	31	34	0.5	0.5	3.75	4.02
19	57	M	AA	20/70	20/50	41	42	0.9	0.9	13.66	8.9
20	65	M	AA	20/200	CF	38	38	0.95	0.95	12.88	3.56
21	62	M	AA	20/20	20/20	27	27	0.8	0.8	9.31	9.43
22	56	F	AA	20/200	20/40	30	23	0.9	0.3	4.81	1.77
23	69	M	AA	20/25	20/25	16	18	0.7	0.8	12.38	7.67
24	86	F	AA	20/20	20/40	29	29	0.3	0.3	4.09	6.51
25	73	F	C	20/25	20/100	22	23	0.8	0.8	14.88	14.3
26	72	M	AA	20/25	20/25	24	28	0.2	0.8	3.29	11.76
27	69	F	AA	20/20	20/25	15	14	0.95	0.95	10.54	11.31

Abbreviations in the table are: Age=age at enrollment; Race=C=Caucasian; AA=African-American; VA=visual acuity; OD=right eye; OS=left eye; Max IOP=maximum IOP measured at Wills Eye Hospital; C/D=vertical cup to disk ratio; HVF PSD=Humphrey visual field pattern standard deviation.

**Figure 2:**
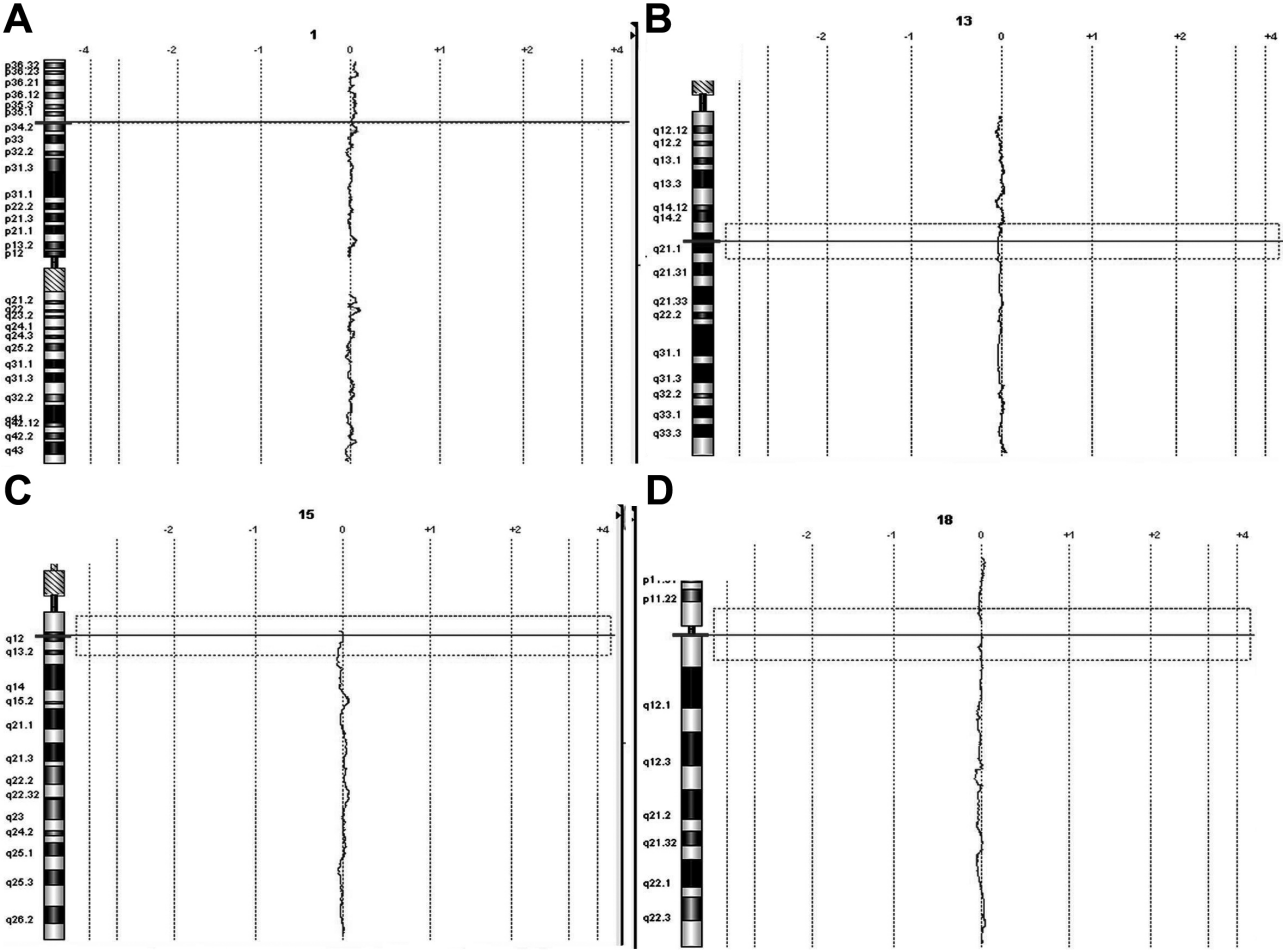
Array CGH results for POAG patients versus controls. Chromosomes shown were chosen randomly as representative of all chromosomes and in all POAG patients tested. In the image, **A** indicates Chromosome 1; **B** indicates Chromosome 13; **C** indicates Chromosome 15; and **D** indicates Chromosome 18. When control DNA was hybridized against POAG DNA, a signal ratio of zero (0) was obtained, indicating the absence of chromosomal copy number alterations.

The signal ratio of each patient compared to a simultaneously tested control (patient-cy3/control-cy5) documented the absence of chromosomal copy number variations in any patient. No POAG patient had evidence of somatic mosaicism. Representative images of array CGH results are shown in Figure 2.

## Discussion

The 27 patients reported here met rigorous clinical criteria for definite POAG [14-16] with elevated IOP, normal anterior chamber, and evidence on the fundoscopic exam and visual fields of glaucomatous optic nerve damage. By clinical criteria, they did not have evidence of other types of glaucoma or alternative causes of optic nerve injury. None had dysmorphism or an obvious genetic syndrome. They were compared to 12 controls in which POAG and other evidence of optic nerve damage were carefully excluded.

High resolution array CGH used here provides quantitative information about the level of chromosome gain or loss such as regions with a high level amplification or high magnitude deletion and will recognize a chromosomal duplication or deletion of a size greater than or equal to 6 Kb. This technique did not detect any chromosomal copy number variations of this size in POAG patients or controls. These results indicate that it is very unlikely that chromosomal deletions or duplications are universally responsible for isolated POAG. Because of the relatively small sample size, it remains possible that chromosomal aberrations might be present in a portion of patients with isolated POAG. More patients from multiple centers and various ethnicities would need to be examined to make a general statement about the absolute absence of chromosomal copy number variations in the setting of POAG. No comment can be made about other chromosomal imbalances such as translocations, inversions, and some ploidies because these cannot be detected by current array CGH technology.

These negative results stand in contrast to reports of chromosomal anomalies causing glaucoma in association with a variety of genetic syndromes and abnormalities of globe development. For example, several chromosomal anomalies have been reported to cause the Axenfeld-Rieger syndrome (OMIM 602482) with variable ocular dysgenesis associated with short height, stunted development of mid-facial features, and mental deficiencies. These anomalies include distal deletions of chromosome 6p [12], duplications [17], balanced translocations [18], and unbalanced translocations [19], but they are all associated with abnormal development of the anterior segment and early onset glaucoma [20,21]. Similarly, one reported patient was documented to have partial trisomy of 7q and partial monosomy of 15q, and the Silver-Russell phenotype (OMIM 180860; low birth weight, delayed maturation, facial dysmorphism, clinodactyly, ivory epiphyses, etc.) with congenital glaucoma [13] while another had a balanced translocation t(9/17)(q34.1;q25) and the Nail-Patella Syndrome (OMIM 161200; dysplasia of the nails, absent or hypoplastic patellae, and a low frequency of glaucoma and ocular hypertension) [22]. Several reported patients with early onset glaucoma and genetic syndromes lack a firm genetic diagnosis [23,24], and micro-anomalies of chromosomes remain possible in some of these patients. The cohort reported here had isolated POAG beginning in late adult life without ocular malformations, dysmorphic features, or other evidence of genetic syndromes, and to date, no such patient has been reported to have an associated chromosome aberration.

In summary, we used high resolution array CGH to evaluate a group of patients with isolated POAG and found no evidence of chromosomal copy number variations. Therefore, neither autosomal genetics [11] nor chromosomal deletions/duplications currently provide a complete explanation for the substantial familial association widely recognized in POAG [2,5]. Although unrecognized genetic or epigenetic factors remain possible, POAG patients do have a variety of mitochondrial [25] and metabolic [26] abnormalities that might put the optic nerve at risk. In this regard, POAG may have certain similarities to Leber hereditary optic neuropathy, another spontaneous optic neuropathy with no obvious autosomal or chromosomal [27] cause that also is associated with mitochondrial abnormalities [28-30].
